# Differential scanning calorimetry study of glycerinated rabbit psoas muscle fibres in intermediate state of ATP hydrolysis

**DOI:** 10.1186/1472-6807-7-41

**Published:** 2007-06-24

**Authors:** Timea Dergez, Dénes Lőrinczy, Franciska Könczöl, Nelli Farkas, Joseph Belagyi

**Affiliations:** 1 Institute of Bioanalysis Faculty of Medicine, H-7624 Pécs, Szigeti str. 12, Hungary; 2 Institute of Biophysics, Faculty of Medicine, H-7624 Pécs, Szigeti str. 12, Hungary; 3 Institute of Forensic Medicine University Pécs, Faculty of Medicine, H-7624 Pécs, Szigeti str. 12, Hungary

## Abstract

**Background:**

Thermal denaturation experiments were extended to study the thermal behaviour of the main motor proteins (actin and myosin) in their native environment in striated muscle fibres. The interaction of actin with myosin in the highly organized muscle structure is affected by internal forces; therefore their altered conformation and interaction may differ from those obtained in solution. The energetics of long functioning intermediate states of ATP hydrolysis cycle was studied in muscle fibres by differential scanning calorimetry (DSC).

**Results:**

SETARAM Micro DSC-II was used to monitor the thermal denaturation of the fibre system in rigor and in the presence of nucleotide and nucleotide analogues. The AM.ADP.P_i _state of the ATP hydrolysis cycle has a very short lifetime therefore, we mimicked the different intermediate states with AMP.PNP and/or inorganic phosphate analogues V_i _and AlF_4 _or BeF_x_. Studying glycerol-extracted muscle fibres from the rabbit psoas muscle by DSC, three characteristic thermal transitions were detected in rigor. The thermal transitions can be assigned to myosin heads, myosin rods and actin with transition temperatures (T_m_) of 52.9 ± 0.7°C, 57.9 ± 0.7°C, 63.7 ± 1.0°C. In different intermediate states of the ATP hydrolysis mimicked by nucleotide analogues a fourth thermal transition was also detected which is very likely connected with nucleotide binding domain of myosin and/or actin filaments. This transition temperature T_m4 _depended on the mimicked intermediate states, and varied in the range of 66°C – 77°C.

**Conclusion:**

According to DSC measurements, strongly and weakly binding states of myosin to actin were significantly different. In the presence of ADP only a moderate change of the DSC pattern was detected in comparison with rigor, whereas in ADP.P_i _state trapped by V_i_, AlF_4 _or BeF_x _a remarkable stabilization was detected on the myosin head and actin filament which is reflected in a 3.0 – 10.0°C shift in T_m _to higher temperature. A similar effect was observed in the case of the nonhydrolyzable AMP.PNP analogue. Differential DSC measurements suggest that stabilization actin structure in the intermediate states of ATP hydrolysis may play an additional role in actin-myosin interaction.

## Background

Force generation in muscle during contraction arises from direct interaction of the two main protein components of the muscle, myosin and actin. The process is driven by the energy liberated from the hydrolysis of ATP. In the presence of Ca^2+^, the energy released from ATP hydrolysis produces conformational changes in myosin and actin, which can be manifested as an internal motion of the myosin head while bound to actin [1-9]. The interaction results in a strain in the head portion of myosin in an ATP-dependent manner and the structural changes lead to a large rotation of the myosin neck region relieving the strain.

The powerful differential scanning calorimetry (DSC) technique allows the derivation of heat capacity of proteins as a function of temperature. From decomposition of the thermal unfolding patterns, it is possible to characterize the structural domains of macromolecules [10-12]. Using the advantage of the SETARAM microcalorimeter in this work, we tried to approach the temperature-induced unfolding processes in different intermediate states of ATP hydrolysis in striated muscle fibres. The main proteins in fibre system are subjected to stabilizing forces to maintain the ability of fibres to contract and to keep the organized structure which can modify the thermodynamic properties of the composite proteins. We studied the fibre system prepared from psoas muscle of rabbit in rigor, strongly binding and weakly binding states of myosin to actin. In the weakly binding state the inorganic phosphate (P_i_) was substituted by the phosphate analogue orthovanadate, AlF_4 _and BeF_x_.

## Results

### Evaluation of thermal transitions in muscle fibres

**Figure 1:**
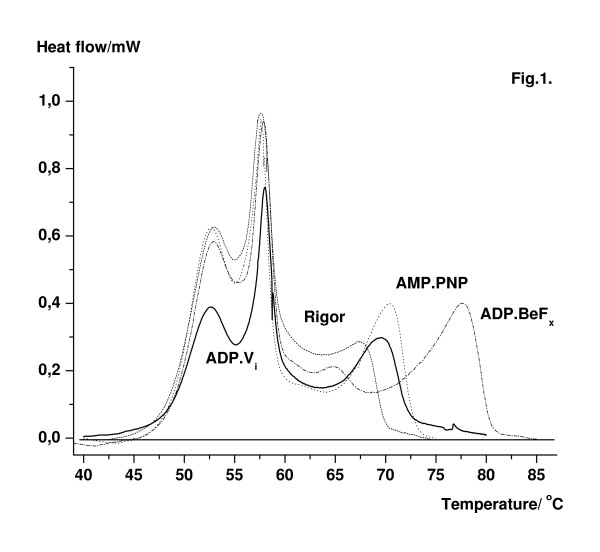
DSC transition curves of muscle fibres in different intermediate states of the ATP hydrolysis (Rigor (dashed line), ADP.V_i _(solid line), AMP.PNP (dotted line), ADP.BeF_x _(dash-dot line)). The melting temperature of the last transition depends strongly on the ligand occupied by the nucleotide binding sites of myosin and/or actin. The heat flow measured in mW is plotted against temperature after baseline correction. The middle transition is not affected by the nucleotides and nucleotide analogues.

The reversibility of heat transition was checked by comparing the first scan of the muscle fibres with the second one after cooling the sample to room temperature. The DSC transitions were calorimetrically irreversible. The first DSC trace was corrected for the calorimetric baseline by subtracting the second scan from the first one and for the difference in heat capacity by using a linear or a sigmoidal baseline. Fig. 1 shows the DSC traces of the different intermediate states (rigor, ADP.V_i_, AMP.PNP, ADP.AlF_4_). Heat flows are plotted as the function of temperature, because our SETARAM micro DSC-II system is a heat flow calorimeter. After the irreversible denaturation of our samples, we could not observe any increase of baseline (that is of the heat capacity, because heat flow divided by heating rate gives this parameter), thus we can exclude the possibility of aggregation process in muscle fibres.

The simplest model, which can be applied for irreversible transitions, was suggested by Lumry and Eyring [13], and applied by Sanchez-Ruiz and co-workers [14-16]. The theory assumed a reversible unfolding of the proteins that is followed by a rate-limiting irreversible step:

N↔k1D→k2F,
 MathType@MTEF@5@5@+=feaafiart1ev1aaatCvAUfKttLearuWrP9MDH5MBPbIqV92AaeXatLxBI9gBaebbnrfifHhDYfgasaacH8akY=wiFfYdH8Gipec8Eeeu0xXdbba9frFj0=OqFfea0dXdd9vqai=hGuQ8kuc9pgc9s8qqaq=dirpe0xb9q8qiLsFr0=vr0=vr0dc8meaabaqaciaacaGaaeqabaqabeGadaaakeaacqqGobGtdaGd0aWcbaGaee4AaS2aaSbaaWqaaiabigdaXaqabaaaleqakiaawsziaiabbseaenaaoqcaleaacqqGRbWAdaWgaaadbaGaeGOmaidabeaaaSqabOGaayPKHaGaeeOrayKaeiilaWcaaa@391F@

where N, D as well as F stand for the native, denatured and final state of the protein, the extent of the irreversibility is determined by the rate constant k_2 _of the D → F step, and its activation energy determines gives the rate of unfolding. Recently, it was shown that in some cases the thermodynamic parameters could be deduced from the standard treatment of the heat capacity curves [17].

**Figure 2:**
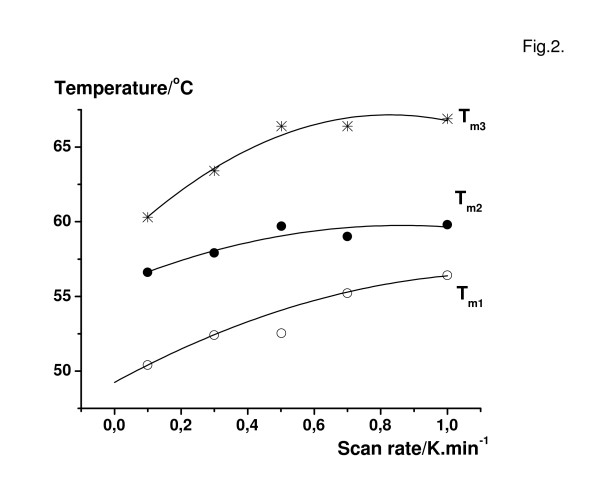
Effect of scanning rate on the DSC transition temperature of glycerol-extracted muscle fibers prepared from m. psoas of rabbit. Measurements were made on fibres in rigor. Figure shows the transition temperatures of the three main transitions. Over 0.5 min.K^-1 ^scan rate the transition temperatures achieve approximately their limiting value. Second-order polynomial functions were used to obtain optimal fitting.

According to the theory, the transition temperatures depend on scan rate, therefore the melting of the muscle samples was determined by kinetic processes and could be described by the two-state irreversible kinetic model (Fig. 2). Second-order polynomial function T_m _= a_0 _+ a_1_s_r _+ a_2_(s_r_)^2 ^was used to obtain optimal fitting by a least squared approximation, s_r _means the scan rate, a_0_, a_1_, a_2 _are constants and T_m _is the measured transition temperature.

**Figure 3:**
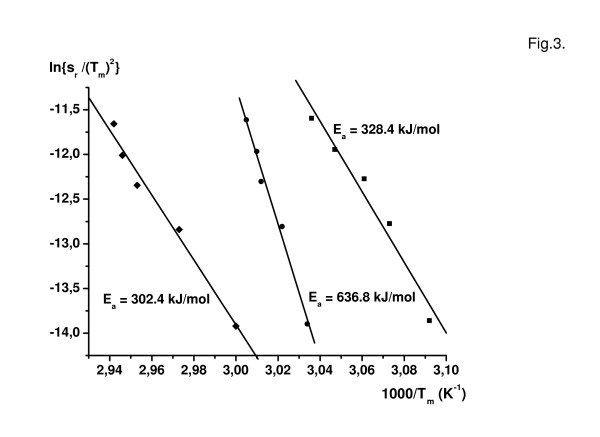
Values of ln{s_r_/(T_m_)^2^} taken from the experiments on rigor fibres are plotted against 1000/T_m_. From the slope of the straight lines the average activation energies can be calculated. Note that a very large value was obtained for the second transitions which can be assigned to the myosin rod.

The kinetic theory predicts that the rate of the denaturation is determined by a first-order rate constant k_2_. The rate constant changes with temperature according to an Arrhenius equation. The expression ln {s_r_/(T_m_)^2^} from measured data on rigor muscle as a function of the reciprocal absolute temperature of T_m _gives a straight line, where s_r _is the scan rate and T_m _means the transition temperature (Fig. 3). From the plot the average activation energies could be obtained for the three main transitions, they were 302, 636 and 328 kJ/mol. The energy of activation corresponds to the irreversible step in the thermal denaturation process. According to data in literature the value of the activation energy of protein denaturation varies between 280–360 kJ/mol, almost independent of the nature of proteins [14,15]. Exceptions are bacteriorhodopsin and annexin V E17G with 731 kJ/mol and 611 kJ/mol, respectively [17]. The middle transition which can be assigned to the myosin rod has the largest activation energy. Very likely, this can be explained by the compact helical structure of the rod. The narrow line width at half height is the sign of the strong cooperative interaction between the molecules in rod structure.

The scan rate practically reached its maximum value at about 0.5 K.min^-1^, therefore this finding permitted the derivation of the thermodynamic equilibrium parameters [17] and the application the decomposition of complex DSC patterns with Gaussian functions. The deconvolution of DSC pattern resulted in three main transitions in rigor; but in different intermediate states a fourth transition at higher temperature was also detected. The first three transitions at lower temperatures were almost independent of the intermediate state of the muscle. The last transition temperature T_m4 _was shifted to higher temperature when the buffer solution was manipulated to mimic the intermediate states of ATP hydrolysis (Table 1, Fig. 1). The mean values of the first three transitions were T_m1 _= 52.9 ± 0.7°C (n = 9), T_m2 _= 57.9 ± 0.7°C (n = 10), T_m3 _= 63.7 ± 1.0°C (n = 7). It is known that the pH of the Tris·HCl buffer has a strong dependence on temperature; therefore, the same measurements were performed using MOPS as an appropriate buffer in order to compare the possible shifts of the transition temperature. The mean values were T_m1 _= 53.2 ± 0.7°C (n = 8), T_m2 _= 57.3 ± 1.0°C (n = 8), T_m3 _= 63.3 ± 1.0°C (n = 4). But similar to Tris·HCl buffer, significant shifts appeared only in the last transition in MOPS buffer as well.

**Table 1:** DSC results on muscle fibres: transition temperatures in range of 40 – 85°C. Glycerinated muscle fibres prepared from psoas muscle of rabbit were measured in rigor, strongly and weakly binding state of myosin to actin. The transition temperatures were derived from the original DSC thermograms

Experimental results	T_1 _(°C)	T_2 _(°C)	T_3 _(°C)	T_4 _(°C)
Rigor	52.2	59.2	-	66.1
ADP	53.0	57.8	-	67.8
AMP.PNP	52.7	57.7	62.1	70.4
PP_i_	52.8	57.5	-	65.8
ADP.V_i_	53.6	57.8	63.3	68.5
ADP.AlF_4_	52.9	57.9	64.4	74.7
ADP.BeF_x_	53.2	58.4	63.8	77.8

Mean values: T_m1 _= 52.9 ± 0.5°C (n = 9)

T_m2 _= 57.9 ± 0.5°C (n = 9)

T_m3 _= 63.7 ± 0.7°C (n = 7)

### Thermal transitions in rigor muscle

Earlier measurements performed on myosin showed that at least five endothermic transitions could be observed on bovine heart myosin. The peak maxima were at 17.5, 41.5, 45, 48 and 54.5°C; the estimated transition enthalpies were 150 kcal/mol, 163 kcal/mol, 277 kcal/mol, 301 kcal/mol and 747 kcal/mol [18,19], but no assignment to the peaks was reported. Slightly different values on S1 were derived by Levitsky's group [20]. As regards the DSC profile of the second main protein in striated muscle, the actin, it is known from literature that DSC measurements on isolated actin showed a single thermal transition. Its melting temperature varied in the range of 63°C and 70°C, depending on the form of actin and the method used in the experiments [21-23]. Structure stabilization of actin filaments in solution by phalloidin and/or jaspakinolide significantly affected the transition temperature as well [24]. Their effects propagated cooperatively along the actin filaments [25]. The contribution of other components of the thin filament, tropomyosin and troponin, to the total enthalpy of denaturation can be neglected in first approximation, because their estimated w% is smaller than 10 w% of the total protein content [26]. Experiments performed on isolated tropomyosin in solution also showed a single sharp thermal transition at 41°C. Tropomyosin in complex with F-actin had no effect on the thermal unfolding of F-actin [27].

In the case of muscle fibres, the constraint generated by filament association and protein-protein interaction increases the structural stability of the supramolecular structure, and it results in larger thermal stability of the system. The structure formation alters the dynamic and energetic properties of the contractile proteins, the consequence of that is the shift of the melting points measured in solution on intact myosin from rabbit psoas muscle to higher temperatures in rigor [28]. This increase of transition temperatures is evidence that particular regions of myosin and actin are subjected to stabilizing forces in the filament system leading to alteration of the transition temperatures. Moreover, the generated internal forces might induce changes in the domain-domain interactions and communications as well. However, it is suggested that during domain-domain and protein-protein interaction, only the protein with a lower transition midpoint is affected by the others possessing a higher transition temperature [29]. In the case of acto.S1 complex, S1 unfolded at much lower temperature than F-actin, but the molecules of S1 remained bound to F-actin even after their full denaturation and thereby S1 could affect the thermal stability of F-actin [30].

### Effect of nucleotides on thermal transitions

**Figure 4:**
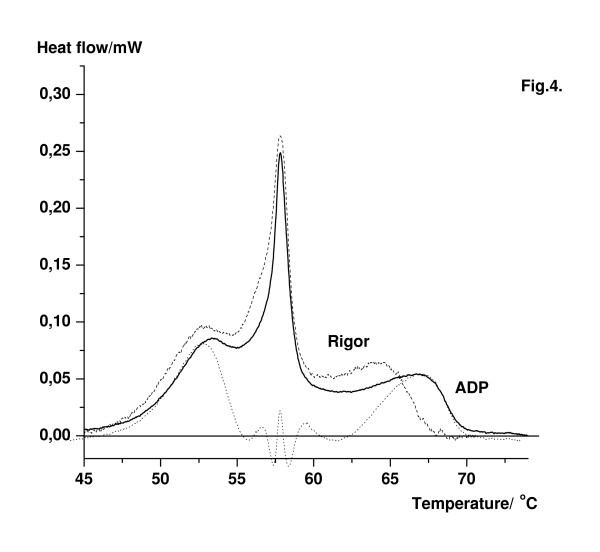
DSC transitions of muscle fibres in rigor (dashed line) and in the presence of ADP (solid line). MOPS buffer was used to minimize the effect of pH change due to increasing temperature. 4 mM MgADP was added to rigor buffer before measurement. In order to demonstrate the effect of MgADP on fibres, the DSC traces of myosin rod and the population of actin at about 64°C were subtracted (dotted line).

Melting curves of glycerol-extracted muscle fibres, detected in strongly binding states of myosin to actin, were indistinguishable in rigor and in ADP state using Tris·HCl buffer. In contrast with this experiment, the addition of 4 mM MgADP to MOPS buffer shifted the last transition temperature T_m4 _to higher temperature with about 2°C from 64.2°C to 66.7°C (Fig. 4). Using the deconvolution procedure of the Peak-Fit software, the single heat transition profiles of myosin rod and actin obtained after manipulation were subsequently subtracted from the experimental DSC pattern to observe the residual effect of proteins. In order to obtain reasonable residuals the melting temperatures of the transitions, the contributions of the individual Gaussian curves to the total endothermic transition and their line widths at half-height of the transition temperature were varied during the manipulation. According to former experiments ADP can bind to F-actin as well. However, it was earlier reported that binding of ADP to F-actin destabilizes the filament. It suggests that ADP-induced alteration in the actin structure cannot produce additional increase in transition temperature. Therefore, we believe that at least one part of the peak at to 66.7°C could be assigned to the nucleotide binding domain of myosin.

## Discussion

Spectroscopic measurements based on spin labelling EPR technique and fluorescence resonance energy transfer showed little differences between the rigor and the ADP states in muscle fibres [31,32]. Maleimide or isothiocyanate spin labels bound to Cys 707 residue of myosin head near to the ATP binding site showed almost identical rotational correlation time in millisecond time range in the saturation transfer EPR time domain in both states. However, the orientation distribution of the attached labels with respect to the longer axis of the fibres was different. This supports the view that the specific binding of ADP to myosin produces a conformational change in the environment of the nucleotide-binding site, and seemingly, this does not lead to overall dynamic change of the motor domain. In contrast, DSC measurements support the view that ADP binding induces global conformational change that leads to structure stabilizing effect in the fibre system.

**Figure 5:**
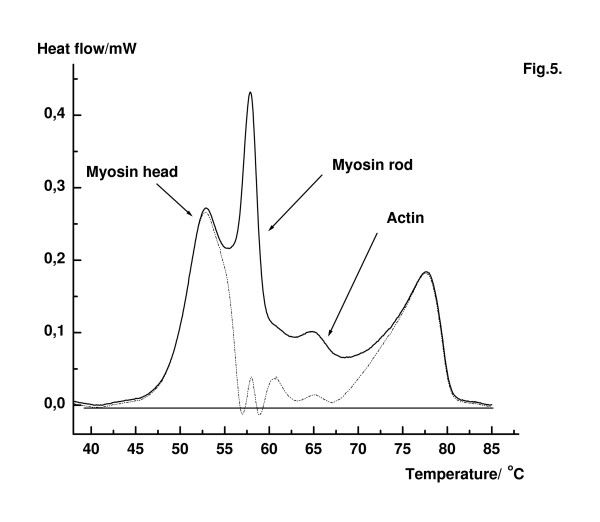
DSC transitions of muscle fibres in the presence of ADP and BF_x _(solid line). DSC traces of myosin rod and the actin population at about 65°C were subtracted (dotted line), using the single Gaussian contributions from the deconvolution. The residual curve shows the transitions of myosin domains and that actin population affected by the nucleotide analogue. Fibre bundle was reacted in a medium containing 4 mM ADP plus 4 mM BeFx in rigor buffer for 15 min before DSC measurement was taken.

Recently, papers published by Levitsky's group reported that in the presence of ADP and inorganic phosphate analogues (BeF_x_, AlF_4 _and V_i_) a large shift of the transition temperature was detected during thermal unfolding of both S1 and F-actin in solution [30,33]. Muhlrad and co-worker have also shown in biochemical experiments based on mild heat treatment, that the structural change that takes place specifically in the 50 kDa region of S1 leads to the loss of ATPase activity and the changed tryptic sensitivity of S1. Nucleotides, ATP or ADP made the 50 kDa region more resistant to heat treatment [34]. The half-life of the myosin.ADP.AlF_4 _complex without actin is 2 days. The presence of actin accelerates decomposition of the complex in solution, and the half-life decreases to 100 min at 25°C [35], but the fibre system remains in relaxed state. The comparison between the DSC patterns in different weakly binding states of myosin to actin in fibre bundles showed that the transition temperature at the fourth (T_m4_) transition varied significantly, depending on the inorganic phosphate analogue, but in the presence of 10 mM PP_i _almost no change was detected in the DSC profile (Table 1). Orthovanadate, aluminium fluoride or beryllium fluoride with ADP affected the fourth (T_m4_) transition in increasing order (Figs 1 and 5). These changes in DSC pattern of the muscle fibre system are due to either to the motor domain of myosin and/or the binding of ADP plus BeF_3 _or AlF_4 _to the nucleotide binding site of F-actin. Precise x-ray experiments led to the conclusion that addition of inorganic phosphate analogues with ADP to myosin altered the structure of the nucleotide binding domain [7,8]. Using our DSC data obtained after deconvolution, the single Gaussian curves were subsequently subtracted from the original thermal transition of fibres. As an approach we assumed that in relaxed state of fibres only a little interaction might exist between myosin heads and the portion of actin showing the thermal transition at about 62–67°C. Moreover, the thermal transition of rods is not involved in the interaction of myosin heads and ADP.BeF_x_. The residual curve (dotted line in Fig. 5) may reflect the contributions from myosin heads with nucleotide analogues and a second portion of actin affected by ADP.BeF_x_.

EPR measurements on spin-labelled myosin in muscle fibres at room temperature showed that the ordered structure in weakly binding state of myosin to actin, which can be recorded in rigor, disappeared and the rotational correlation time of myosin heads was significantly reduced [36]. The rotational correlation time of about 1 ms in rigor decreased with about one order of magnitude calculated from ST EPR spectra. This mobility change suggests the dissociation of myosin heads from actin or the existence of a non-specific binding of myosin heads to actin, and these actin-bound heads are mobile in the weakly binding state [37]. The mean orientation of myosin heads to actin filaments is about 90°, and a large effect of such heads to the actin dynamics is not expected.

**Figure 6:**
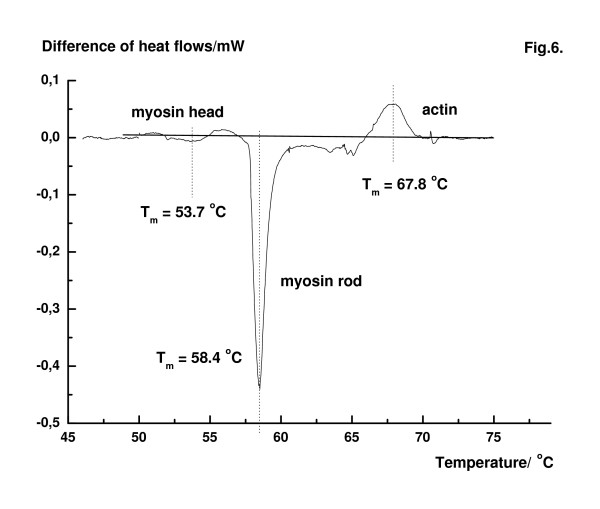
Difference of heat flow of muscle fibers in rigor. Myosin was partially extracted from one of the fibers. The fibers in the two sample holder had exactly the same weight with an accuracy of ± 1 mg. After myosin extraction the fibres had relatively more actin in comparison with untreated fibers.

In order to make a reliable decision, differential measurements were performed. The special construction of the SETARAM Micro-DSC II with two identical sample holders allows the simultaneous measurement of two muscle samples. The output current of amplifiers in this case gives the difference of the heat flows. Myosin was partially extracted from one of the fibre bundle and then it was measured against an untreated fibre bundle in the same scan. Following the myosin extraction procedure the weights of the control and treated fibre bundles were so adjusted that they had equal weights in the sample holders with an accuracy of ± 1 mg. As a result of this manipulation the treated fibre bundles contained relatively more actin in contrast with the control bundle which had more myosin. The measurement obtained in rigor is shown in Fig. 6. The DSC pattern of the untreated fibre bundle shows a small extra transition at 53.7°C which comes from excess myosin. The peak of the treated fibre bundle at 67.8°C in the DSC profile originates from the excess of actin. The middle peak refers to the larger amount of myosin in the untreated fibre bundle (58.4°C). The analysis based on these differences suggests the view that the last transition in rigor fibres at about 63–68°C can be accounted for the unfolding of F-actin filaments.

**Figure 7:**
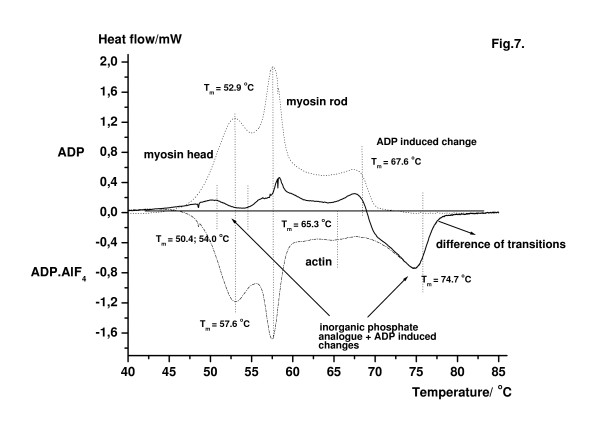
Difference of heat flows by computer manipulation. The solid line was obtained as a difference of heat flows from two original DSC patterns. The samples were differently treated. Dotted line shows the DSC pattern from muscle fibres after ADP treatment, whereas dash-dot line is the DSC pattern obtained after ADP.AlF_4 _treatment. No difference could be detected at the region of myosin rods. The largest effect appeared in the thermal transition region of thin filaments.

This new procedure can be demonstrated by computer manipulation. The difference of heat flows was prepared from two original DSC patterns (Fig. 7). The samples were differently treated. Dotted line shows the DSC pattern from muscle fibres after 4 mM ADP treatment, whereas dash-dot line is the DSC pattern obtained after 15 min ADP.AlF_4 _treatment. Solid line shows the difference. From the artificially made difference it can be concluded that addition of ADP produced about 2°C increase of thermal transition of actin. In the intermediate state (ADP.AlF_4_) a more remarkable change could be derived. The largest effect was obtained in the transition region of F-actin, and a much smaller change could be observed in the region of myosin head. But almost no difference could be measured in the temperature range of thermal transition of myosin rods.

**Figure 8:**
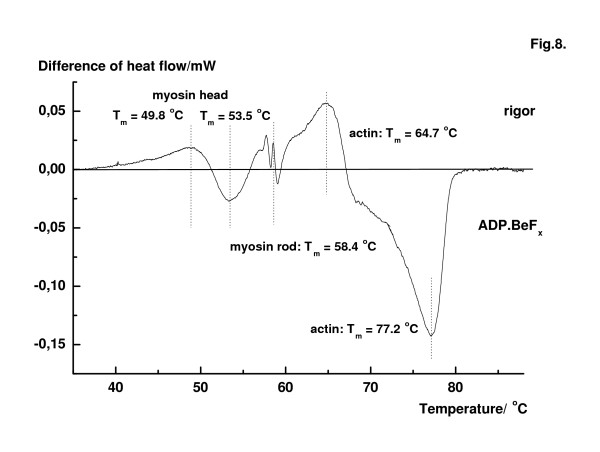
Comparison of heat flows of muscle fibres in rigor and ADP.BeF_x _state. The fibres in the two sample holder had exactly the same weight with an accuracy of ± 1 mg. Note the large shift of actin transition from 65°C to 77.4°C and the lack of peak at about 58°C.

Differential measurement was also performed on fibre bundles which were treated differently before DSC measurements. Typical result is demonstrated in Fig. 8. Fibre bundles in rigor and in the ADP.BeF_x _states were denatured against each other in the same run. The bundles had equal weights. The main protein components of the fibres were affected by ATP plus BeF_x_. The transition temperature of myosin heads shifted with about 4°C in the ADP.BeF_x _state (53.5°C) in comparison with the myosin heads in rigor, while the denaturation temperature of the F-actin filaments increased more than 10°C in the ADP.BeF_x _state. Almost no excess heat absorption can be observed at 58.3°C in the ADP.BeF_x _state, which is the transition temperature of the myosin rods. This support the suggestion that the thermal transition of myosin rod is not affected by nucleotides and nucleotide analogues bound to myosin head.

In order to interpret the DSC results, we might adopt the suggestion by Hozumi and Bombardier and co-workers about the existence of a second nucleotide binding site on F-actin [38,39]. Unfortunately, little is known about the properties and the role of the second nucleotide binding site on actin. Based on former observations, it is possible to give alternative explanations. The binding of nucleotides or nucleotide analogues would affect the stability of thin filaments against heating. Carlier and co-workers reported that phosphate release in actin was reversible; P_i _could rebound to actin by producing F-actin-ADP.P_i _filament, which is more stable than ADP-F-actin [40]. Orlova and Egelman [41,42] have also shown by EM that beryllium fluoride and phosphate made the flexible filaments rigid which may support an induced communication between the nucleotide binding site and other region of actin protomers. The perturbations spread to neighbouring protomers and thereby affect the dynamic nature of F-actin [43]. This increased stability of F-actin filaments could lead to increased heat absorption.

The second possible explanation of the DSC experiments might relay on the assumption that F-actin molecules in fibres could form two populations in the presence of nucleotides and nucleotide analogues: (i) protomers with MgADP or MgADP-P_i _analogue in the nucleotide cleft, which distributed randomly on the filaments, or (ii) MgADP protomers in the nucleotide cleft and protomers with MgADP – P_i _analogue bound to the second nucleotide binding sites. The binding of V_i_, AlF_4 _or BeF_x _to F-actin increases the stability of filaments and leads to the shift of T_m3_. The fourth heat transition T_m4 _depended on the bound nucleotides as shown by Figs 1 and 5 and Table 1. The largest effect was detected in the case of BeF_x_, and only a small effect was obtained in the presence of P_i_. DSC measurements performed on F-actin solution support the view that BeF_x _and a lesser extent AlF_4 _stabilize F-actin, and they protect it from heat denaturation [44,45]. It cannot be excluded that there is a possibility that the increased stability of actin filaments in the presence of ATP and inorganic phosphate during contraction might have significance in the force generation [46]. Recent DSC experiments do not support this assumption. The hydrolysis product P_i _can bind to ADP-actin subunits and the newly formed ADP.P_i_-actin subunits dissociates much slowly from filament ends than ADP-actin subunits [40]. So, the change in filament flexibility is affected by the ADP.P_i_-subunits at the end of filaments which might produce an additional stability in the muscle machine. However, DSC measurements showed little effect in the presence of P_i_, therefore only a minor effect is expected in contrast to the increased filament stability evoked by inorganic phosphate analogues as V_i_, AlF_4 _or BeF_x_.

## Conclusion

(i) The DSC experiments carried out on muscle fibres are an extension of former measurements performed on isolated motor proteins (actin and myosin) and their complexes in solution. The approximation of the muscle motor system in our experiments takes into account the specific interaction between the basic motor proteins in highly organized system that allows cyclic motion in the presence of ATP.

(ii) The thermal transitions are calorimetrically irreversible in the muscle fibre system as well, therefore the DSC traces are kinetically controlled and scan-rate dependent, and the application of theory of irreversible thermal denaturation is required. The experienced scan-rate effect permitted the decomposition of the DSC traces into separate transitions and the assignment to protein subunits. In both strongly and weakly binding states of myosin to actin, nucleotides and nucleotide analogues affected specifically the thermal transitions of the myosin heads.

(iii) In order to assign the transitions to the macromolecular components, a *differential method *was introduced for interpretation of DSC data. The analysis showed the role of actin in this complex system containing extra ADP.P_i _analogues, as ADP.V_i_, ADP.AlF_4 _or ADP.BeF_x_.

## Methods

### Chemicals

ADP, ATP, 5'-adenylyl-imido-diphosphate (AMP.PNP), aluminium chloride, beryllium sulphate, P1, P5-di (adenosine-5') pentaphosphate, EGTA, glycerol, lactic dehydrogenase, 4-morpholinepropanesulfonic acid (MOPS), phosphoenol pyruvic acid, pyruvate kinase and sodium fluoride were obtained from Sigma (Germany).

### Fibre preparation

Glycerol-extracted muscle fibre bundles were prepared from rabbit psoas muscle as described earlier [47]. Strong actin binding state to myosin (AM.ADP), as well as weak actin binding transition state (AM.ADP.P_i_) was monitored. In experiments involving MgADP, the activity of adenylate kinase was inhibited by the addition of 50 μM diadenosine pentaphosphate. AM.ADP.P_i _state was mimicked by addition of ATP and beryllium or aluminium fluoride or orthovanadate. Beryllium fluoride and aluminium fluoride were prepared from 10 mM NaF and 3 mM AlCl_3 _and BeSO_4 _immediately before experiments. Muscle fibres were stored in solution containing 80 mM KPr, 5 mM ATP, 5 mM MgCl_2_, 1 mM EGTA in 20 mM MOPS pH 7.0 plus the corresponding chemicals for 15 minutes at 0°C and then a DSC measurement was taken. The fibre bundles prepared in this way were able to develop tension and they could repeatedly shorten and relax in a suitable buffer solution.

### Manipulations with fibre preparations

Myosin was extracted from fibres in a solution containing 0.3 M KCl, 0.15 M K-phosphate, 2 mM EDTA and 1 mM ATP, pH 6.5 at 0°C for 15–20 min before DSC measurements.

### ATPase activity

The ATPase activity was determined using a pyruvate kinase-lactate dehydrogenase coupled optical test [48,49] as described in an earlier paper [47]. The decrease of absorbance at 340 nm resulted in a straight line, and from its slope the ATPase activity was estimated. The Mg^2+^-ATPase activity (μmmole of P_i_/mg myosin.min) was 4.131 ± 0.718 μmmole of P_i_/mg myosin.min (n = 4). The ATPase activity of active fibre bundles was 5.565 ± 0.816 μmmole of P_i_/mg myosin.min (n = 4).

### DSC technique

Thermal unfolding of muscle proteins in different states was monitored by a SETARAM Micro DSC-II calorimeter. Conventional Hastelloy batch vessels were used with 850 μL sample volume (muscle fibres plus buffer) in average. Typical muscle wet weights for calorimetric experiments were between 200 – 250 mg. Rigor buffer was used as a reference sample. The sample and reference vessels were equilibrated with a precision of ± 0.1 mg. The repeated scan of denatured sample was used as baseline reference, which was subtracted from the original DSC curve. The decomposition of the heat transition trace of muscle fibres was performed by the method of the 'successive annealing' suggested for the analysis of complex systems, as muscle proteins [20,50]. A heating-cooling-heating cycle up to the subsequent heat transition maximum was repeated to derive the unfolding of the consecutive components of muscle fibres, which have different thermal stability.

### Evaluation of DSC measurements

In strongly and weakly binding states of myosin to actin the thermograms could be decomposed into four separate transitions in the main transition temperature range. Deconvolution into four components was performed by using PeakFit 4.0 software from SPSS Corporation. For analysis of the single thermal transitions, Gaussian functions were assumed. The program allowed the determination of peak centre, full width at half maxima and % area of the single transitions. A square matrix presented the overlap areas between any two peaks. A laboratory developed computer program was used to subtract the single transitions. It was assumed that the different nucleotides and nucleotide analogues do not significantly affect the thermal properties of the myosin rod, and one population of actin is not perturbed by myosin in the weakly binding state of myosin to actin in the first approximation.

## Authors' contributions

TD and NF have carried out the sample preparation, DL has performed the DSC experiments and participated in the design of study and helped to draft the manuscript, FK has helped in the statistical analysis and figure preparation, JB participated in the design of the study and its coordination and helped to draft the manuscript.

## Supplementary Material

Additional file 1 Table 1: DSC results on muscle fibres: transition temperatures in range of 40 – 85°C. The data represent the statistical analysis of DSC measurements. Click here for file

Additional file 2 Figure Legends. The file contain the explanations of Figs presented in our work. Click here for file

## References

[B1] Geeves MA (1991). The dynamics of actin and myosin association and the crossbridge model of muscle contraction. Biochem J.

[B2] Holmes KC (1998). A molecular model for muscle contraction. Acta Crysallogr.

[B3] Holmes KC (1998). A powerful stroke. Nature Struct Biol.

[B4] Geeves MA, Holmes KC (1999). Structural mechanism of muscle contraction. Ann Rev Biochem.

[B5] Rayment I, Holden HM, Whittaker M, Yohn CB, Lorenz M, Holmes KC, Milligan RA (1993). Structure of the actin-myosin complex and its implications for muscle contraction. Science.

[B6] Dominguez R, Freyzon Y, Trybus KM, Cohen C (1998). Crystal structure of a vertebrate smooth muscle myosin motor domain and its complex with the essential light chain: visualization of the pre-power stroke state. Cell.

[B7] Fisher AJ, Smith CA, Thoden J, Smith R, Sutoh K, Holden HM, Rayment I (1995). Structural studies of myosin:nucleotide complexes: a revised model for the molecular basis of muscle contraction. Biophys J.

[B8] Fisher AJ, Smith CA, Thoden J, Smith R, Sutoh K, Holden HM, Rayment I (1995). X-ray structures of the myosin motor domain of *Dictyostellium discoideum *complexed with MgADP.BeF_x _and MgADP.AlF_4_. Biochemistry.

[B9] Pate E, Naber N, Matuska M, Franks-Skiba K, Cooke R (1997). Opening of the myosin nucleotide triphosphate binding domain during the ATP cycle. Biochemistry.

[B10] Privalov PL, Potekhin SA (1986). Scanning microcalorimetry in studying temperature-induced changes in proteins. Methods Enzymol.

[B11] Sturtevant JM (1987). Biochemical applications of differential scanning calorimetry. Ann Rev Phys Chem.

[B12] Zolkiewski M, Redowicz MJ, Korn ED, Ginsburg A (1996). Thermal unfolding of *Acanthamoeba *myosin II and skeletal muscle myosin. Biophys Chem.

[B13] Lumry R, Eyring H (1954). Conformation changes of proteins. J Phys Chem.

[B14] Sanchez-Ruiz JM, Lopez-Lacomba JL, Cortijo M, Mateo PL (1988). Differential scanning calorimetry of the irreversible thermal denaturation of thermolysin. Biochemistry.

[B15] Conjero-Lara F, Mateo PL, Aviles FX, Sanchez-Ruiz JM (1991). Effect of Zn^++ ^on the thermal denaturation of carboxipeptidase B. Biochemistry.

[B16] Thorolfsson M, Ibarra-Molero B, Fojan P, Petersen SB, Sanchez-Ruiz JM, Martinez A (2002). L-Phenylalanine binding and domain organization in human phenylalanine hydroxylase: a differential scanning calorimetry study. Biochemistry.

[B17] Vogl T, Jatzke C, Hinz H-J, Benz J, Huber R (1997). Thermodynamic stability of annexin V E17G: equilibrium parameters from an irreversible unfolding reaction. Biochemistry.

[B18] Lõrinczy D, Hoffmann U, Pótó L, Belagyi J, Laggner P (1990). Conformational changes in bovine heart myosin as studied by EPR and DSC techniques. Gen Physiol Biophys.

[B19] Samejima K, Ishioroshi M, Yashui T (1983). Scanning calorimetric studies on thermal denaturation of myosin and its subfragment. Agric Biol Chem.

[B20] Levitsky DI, Shnyrov VL, Khvorov NV, Bukatina AE, Vedenkina NS, Permyakov EA, Nikolaeva OP, Poglazov BF (1992). Effects of nucleotide binding on thermal transitions and domain structure of myosin subfragment 1. Eur J Biochem.

[B21] Bertazzon A, Tsong TY (1990). Effects of ions and pH on the thermal stability of thin and thick filaments of skeletal muscle: high sensitivity differential scanning calorimetric study. Biochemistry.

[B22] Lõrinczy D, Könczöl F, Gaszner B, Belagyi J (1998). Structutal stability of actin as studied by DSC and EPR. Thermochim Acta.

[B23] Nikolaeva OP, Dedova IV, Khorova IS, Levitzky DI (1994). Interaction of F-actin with phosphate analogues studied by differential scanning calorimetry. FEBS Letters.

[B24] Visegrády B, Lõrinczy D, Hild G, Somogyi B, Nyitrai M (2004). The effect of phalloidin and jaspaklinolide on the flexibility and thermal stability of actin filaments. FEBS Letters.

[B25] Visegrády B, Lõrinczy D, Hild G, Somogyi B, Nyitrai M (2005). A simple model for the cooperative stabilisation of actin filaments by phalloidin and jasplakinolide. FEBS Letters.

[B26] Bagshaw CR (1993). Muscle contraction. Appendix p145–146.

[B27] Levitsky DI, Rostkova EV, Orlov VN, Nikolaeva OP, Moiseeva LN, Teplova MV, Gusev NB (2000). Complexes of smooth muscle tropomyosin with F-actin studied by differential scanning calorimetry. Eur J Biochem.

[B28] Lõrinczy D, Belagyi J (2001). Nucleotide binding induces global and local structural changes of myosin head in muscle fibres. Eur J Biochem.

[B29] Brands JF, Cui Qing Hu, Lung-Nan Lin (1989). A simple model for proteins with interacting domains. Applications to scanning calorimetry data. Biochemistry.

[B30] Levitsky DI, Nikolaeva OP, Orlov VN, Pavlov DA, Ponomarev MA, Rostkova EV (1998). Differential scanning calorimetric studies on myosin and actin. Biochemistry (Moscow).

[B31] Belagyi J, Frey I, Pótó L (1994). ADP-induced changes in ordering of spin-labelled myosin heads in muscle fibres. Eur J Biochem.

[B32] Fajer PG, Fajer EA, Matta JM, Thomas DD (1990). Orientational distribution of crossbridges in muscle fibres in rigor and ADP. Biochemistry.

[B33] Bobkov AA, Khovorov NK, Golitsina NL, Levitsky DI (1993). Calorimetric characterization of the stable complex of myosin subfragment 1 with ADP and beryllium fluoride. FEBS Letters.

[B34] Setton A, Muhlrad A (1984). Effect of mild heat treatment on the ATPase activity and proteolitic sensitivity of myosin subfragment-1. Arch Biochem Biophys.

[B35] Werber MM, Peyser YM, Muhlrad A (1992). Characterization of stable beryllium fluoride, aluminium fluoride and vanadate containing myosin subfragment 1-nucleotide complexes. Biochemistry.

[B36] Raucher D, Fajer EA, Sar C, Hideg K, Zhao Y, Kawai M, Fajer PG (1995). A novel electron paramagnetic resonance spin label and its application to study the cross-bridge cycle. Biophys J.

[B37] Thomas DD, Ramachandran S, Roopnarine O, Hayden DW, Ostap EM (1995). The mechanism of force generation in myosin: A disorder-order transition, coupled to internal structural changes. Biophys J.

[B38] Hozumi T (1988). Structural aspects of skeletal muscle F-actin as studied by tryptic digestion: evidence for a second nucleotide interacting site. J Biochem.

[B39] Bombardier H, Wong P, Gicquaud C (1997). Effects of nucleotides on the denatu-ration of F-actin: a differential scanning calorimetry an FTIR spectroscopy stu-dy. Biochem Biophys Res Comm.

[B40] Carlier M-F, Pantaloni D (1988). Binding of phosphate to F-ADP-actin and the role of F-ADP-P_i_-actin in ATP-actin polymerization. J Biol Chem.

[B41] Orlova A, Egelman EH (1993). A conformational change in the actin subunit can change the flexibility of the actin filament. J Mol Biol.

[B42] Orlova A, Egelman EH (1992). Structural basis for the destabilization of F-actin by phosphate release following ATP hydrolysis. J Mol Biol.

[B43] Muhlrad A, Cheung P, Phan BC, Miller C, Reisler E (1994). Dynamic properties of actin. Structural changes induced by beryllium fluoride. J Biol Chem.

[B44] Combeau C, Carlier M-F (1988). Probing the mechanism of ATP hydrolysis on F-actin using vanadate and the structural analogs of phosphate BeF_3 _and AlF_4_. J Biol Chem.

[B45] Dancker P, Schmied GH (1989). Stabilization of actin filaments by ATP and inorganic phosphate. Z Naturforsch.

[B46] Lombardi V, Piazzesi G, Linari M (1992). Rapid regeneration of the actin-myosin power stroke in contracting muscle. Nature.

[B47] Hartvig N, Lõrinczy D, Farkas N, Belagyi J (2002). Effect of adenosine 5'[β,γ-imido]triphosphate on myosin head domain movements. Eur J Biochem.

[B48] Trentham DR, Bardsley RG, Eccleston JP, Weeds AG (1972). Elementary processes of the magnesium ion-dependent adenosine triphosphatase activity of heavy meromyosin. Biochem J.

[B49] Norby JG (1971). Studies on a coupled enzyme assay for rate measurements of ATPase reactions. Acta Chem Scand.

[B50] Levitsky DI, Khovorov NV, Shnyrov VL, Vedenkina NS, Permyakov EA, Poglazov BF (1990). Domain structure of myosin subfragment-1. Selective denaturation of the 50 kDa segment. FEBS Letters.

