# Supplementary material for: Differential scanning calorimetry study of glycerinated rabbit psoas muscle fibres in intermediate state of ATP hydrolysis
Source: BMC Struct Biol. 2007 Jun 24;7:41. doi: 10.1186/1472-6807-7-41 (PMC1913527; doi:10.1186/1472-6807-7-41)
Supplement: Additional file 1 — Table 1: DSC results on muscle fibres: transition temperatures in range of 40 – 85°C. The data represent the statistical analysis of DSC measurements. [file 1472-6807-7-41-S1.doc]

**T a b l e 1.**: **DSC results on muscle fibres: transition temperatures in range of 40 - 85 ^o^ C.**

Glycerinated muscle fibres prepared from psoas muscle of rabbit were measured in rigor, strongly and weakly binding state of myosin to actin. The transition temperatures were derived from the original DSC thermograms.

| Experimental results | T_1_ (°C) | T_2_ (°C) | T_3_ (°C) | T_4_ (°C) |
| --- | --- | --- | --- | --- |
| Rigor | 52.2 | 59.2 | - | 66.1 |
| ADP | 53.0 | 57.8 | - | 67.8 |
| AMP.PNP | 52.7 | 57.7 | 62.1 | 70.4 |
| PP_i_ | 52.8 | 57.5 | - | 65.8 |
| ADP.V_i_ | 53.6 | 57.8 | 63.3 | 68.5 |
| ADP.AlF_4_ | 52.9 | 57.9 | 64.4 | 74.7 |
| ADP.BeF_x_ | 53.2 | 58.4 | 63.8 | 77.8 |

Mean values: T_m1_ = 52.9 ± 0.5 °C (n =9)

T_m2_ = 57.9 ± 0.5 °C (n =9)

T_m3_ = 63.7 ± 0.7 °C (n =7)
